# The symbolizations emerging from aviation crew members’ perspectives on well-being: a linguistic analysis using emotional text mining

**DOI:** 10.3389/fpsyg.2025.1612940

**Published:** 2025-06-18

**Authors:** Alessia Renzi, Matteo Reho, Manuela Tomai, Micaela Scialanga, Barbara Cordella

**Affiliations:** ^1^Department of Dynamic and Clinical Psychology and Health Studies, “Sapienza” University of Rome, Rome, Italy; ^2^Department of Biomedical, Metabolic and Neural Sciences, University of Modena and Reggio Emilia, Modena, Italy; ^3^IT-APA (Italia-Associazione Psicologia dell’Aviazione), Rome, Italy

**Keywords:** well-being, aviation, aviation crew members, emotional text mining, psychological service

## Abstract

**Background:**

Well-being is a complex and structured construct recently studied in aviation, primarily through self-report measures. The primary aim of this study is to explore aviation crews’ representations of well-being using the Emotional Text Mining (ETM) method. A secondary aim is to examine whether different representations are associated with occupational variables.

**Method:**

492 participants (302 males) completed an online survey via SurveyMonkey, including an open-ended question prompting them to describe their perception of well-being. Additionally, socio-occupational variables were collected. The texts were analyzed using ETM, identifying how a social group emotionally symbolizes a topic.

**Results:**

The analysis revealed three clusters: (1) Material and Family Stability, (2) Individual Self-Development, and (3) Connection and Social Realization. These clusters are positioned within a factorial space defined by two factors: one contrasting individual vs. social representations of well-being and the other differentiating well-being as a set of given elements vs. an active, evolving process. No associations with occupational variables were found.

**Conclusion:**

The findings suggest a concept of well-being in which the work dimension is considered only marginally, primarily for its material and economic value, and not as part of a broader sense of personal fulfillment. Administering the survey within a workplace setting may have heightened social desirability bias, potentially influenced by fears of professional repercussions.

## 1 Introduction

Well-being is a multifaceted and complex concept, with various professionals offering different perspectives, resulting in diverse definitions (Disabato et al., 2016; Tov, 2018). Research in well-being exploring its relationship with job functioning and performance is relatively recent. It pertains to the sense of fulfillment derived from work, encompassing both employees’ overall emotional state and the intrinsic and extrinsic values associated with their jobs, including also employees’ subjective assessment of their ability to develop and function optimally within the work environment (Aryanti et al., 2020; Bartels et al., 2019). Occupational well-being plays a crucial role in employees’ psychophysical health and is a key factor in the long-term sustainability of an organization (Aryanti et al., 2020). Indeed, individuals with higher levels of occupational well-being tend to be more creative and collaborative, exhibit lower turnover rates, and demonstrate greater productivity at work (Miller, 2016; Stephens et al., 2013).

In this light, organizations and consultancy firms have increasingly focused on enhancing employee well-being (Wijngaards et al., 2022). This change has also influenced the aviation sector, especially in the wake of tragic incidents—such as the 2015 Germanwings Flight 9,525 crash in the French Alps and the 2022 China Eastern Airlines disaster—which have highlighted the critical importance of pilots’ psychological well-being in both public discourse and international scientific research (Chang et al., 2024). The aviation industry is characterized by high job demands, constant pressure, and a perfectionist work culture, often blurring professional and personal life (Agarwal et al., 2024; Chang et al., 2024). This dynamic can significantly affect employees’ well-being, influencing their work performance and personal lives (Agarwal et al., 2024; Cahill et al., 2020).

Work-related stress significantly affects pilots’ well-being, leading to issues such as exhaustion, disengagement, burnout, depression, and anxiety (Cahill et al., 2020; Cullen et al., 2021; Demerouti et al., 2019; Wu et al., 2016). A recent study found that role-related stress significantly contributed to emotional exhaustion in pilots, negatively impacting overall well-being (Chang et al., 2024). It has also been found that high levels of workplace involvement and responsibility have a negative association with subjective well-being (Agarwal et al., 2024). In conclusion, organizations’ commitment to enhancing employee well-being remains a critical area requiring further investigation (Söderlund and Sagfossen, 2017), particularly in the aviation sector (Chang et al., 2024; Tang et al., 2020), where operators’ physical and mental health directly impact not only passenger satisfaction but also safety.

In recent decades, the relationship between well-being and work performance has been explored using various methods and approaches (Kansky and Diener, 2017); among them, the most commonly used are self-report instruments (e.g., Bartels et al., 2019; Lucas et al., 1996). However, self-report measures may not fully capture the multifaceted nature of well-being (Arthaud-day et al., 2005; Kansky and Diener, 2017) and are subject to significant biases. Aviation research also predominantly relies on self-report instruments, and only in recent years has it begun to incorporate qualitative methods. Qualitative approaches adopt a holistic perspective that preserves the complexity of human behavior, allowing for the exploration of the rational aspects of expressed experiences and their more profound emotional components (Lakshman et al., 2000; Queirós et al., 2017).

The present study aims to explore: (1) the representations of well-being among crew members through textual analysis methods enabling the identification of collusive processes through which a specific social group emotionally symbolizes a topic (Greco and Polli, 2020); (2) whether certain socio-occupational variables (e.g., age, current role, flight hours) are associated with different recurring themes on well-being. The broader aim is to identify elements functional to enhance the use of the psychological support program called “Support Programme” to sustain the psychological well-being of flight crews (see material and methods).

## 2 Materials and methods

### 2.1 Participants

The survey was promoted by IT-APA Italia – Associazione Psicologia dell’Aviazione and realized in collaboration with the Department of Dynamic and Clinical Psychology and Health Studies, “Sapienza” University of Rome. The University Ethical Committee approved the study (protocol no. 0000328, April 16, 2020). Participation was proposed to pilots (flight crew), the primary professionals targeted by the psychological support program known as the “Support Programme,” as well as to flight attendants (cabin crew). This program, mandated by the European aviation authority EASA – European Aviation Safety Agency (EU Regulation, 2018), is designed to protect or restore the psychological well-being of flight crews through support provided by trained peers and/or aviation psychologists, with the option of referral to external psychological professionals if necessary. Although the regulation requires that the program be implemented specifically for pilots, it also encourages its extension to other safety-sensitive personnel, such as flight attendants and maintenance technicians. For this reason, participation was also proposed to flight attendants. Initial contact was made with 16 operators, but only 3 of them showed strong commitment to the current study by actively inviting their crews to complete the survey. These operators sent official business emails containing detailed information about the study’s objectives and the voluntary, non-incentivized nature of participation. They also distributed a SurveyMonkey link through which participants could provide informed consent and complete the questionnaire. To further promote participation, the study was also presented to trade unions. Additionally, a description of the research and an invitation to participate were shared with the national target population via the IT-APA website and LinkedIn page. A total of 791 responses were collected, of which 299 did not include an answer to the open-ended question on well-being and were therefore excluded from the analysis. This exclusion was necessary because the present study is part of a broader research project which, as described in the measure section outlining the survey questionnaire, includes both closed-ended and one open-ended questions. Some participants responded only to the closed-ended items, which are not relevant to the specific aims of the present study. The final sample included 492 responses: 191 from females and 302 from males (for age ranges, flight hours, and roles, see Table 1).

**Table 1:** Participants’ anamnestic and occupational characteristics.

Variable	Categories/ranges	*N*	%
Age range	≤25	35	7.10
	26–30	32	6.5
	31–35	33	6.7
	36–40	55	11.2
	41–45	89	18.1
	46–50	90	18.3
	51–55	91	18.5
	>56	67	13.6
Current role	Flight attendant	133	27
	Cabin manager	64	13
	Fixed wing captain	130	26.4
	Rotary wing captain	28	5.6
	First officer/Fixed-wing copilot	128	26.1
	First officer/Rotary wing copilot	5	1.1
	Rotary wing technical specialist	4	0.8
Total personal flight hours	0–1,500	58	11.7
	1,501–5,000	49	10
	5,001–10,000	88	17.8
	10,001–15,000	108	21.8
	15,001–20,000	84	17
	20,001–25,000	25	5
	25,001–30,000	8	1.5
	>30,000	12	2.4
	I do not remember	60	12.8

### 2.2 Measures

An *ad hoc* questionnaire collected demographic and professional information about the participants, such as gender, age range, flight hours, and current role (see Table 1).

A specific questionnaire consisting of 11 closed questions, already used in previous research (see Reho et al., 2025; Scialanga et al., 2020), was used to investigate the participants’ opinions on the role of the psychologist, the propensity to seek psychological support, and the conception of well-being. The present study focuses on the final open-ended question aimed at exploring the individual representation of well-being. About the open question, participants were asked: “How would you describe the concept of well-being in a person’s life? We would like to know your idea about it. Write freely without worrying about syntax. We recommend writing at least five lines of text for efficient statistical processing”.

### 2.3 Data analysis

The answers to the open question were transcribed and collected in a single corpus and analyzed through T-Lab software (Lancia, 2012) using the emotional text mining method (ETM), grounded in socio-constructivist epistemology and psychodynamic theoretical principles (Greco and Polli, 2020). ETM facilitates the extraction and interpretation of both semantic structures (content of the communication-conscious level) and latent semiotic configurations (emotional symbolization-unconscious level) embedded within discursive productions (Cordella et al., 2014).

Using the T-LAB software a cluster analysis was performed with emerging clusters representing a discursive nucleus thematically and symbolically coherent. The CA was performed adopting the unsupervised K-Means as clustering method. T-Lab employs an algorithm that iteratively determines the optimal number of clusters. Specifically, the process stops when further partitioning the data does not significantly improve the balance between internal cohesion and separation between groups, indicating that new clusters would not add useful information. The interpretation of the clusters was conducted following a consensus procedure (Harris et al., 2012). Each cluster was labeled independently by members of the research team (all PhD-qualified psychologists) based on the characteristic lemmas within them (Table 2). The team then collectively discussed the proposed labels, resolving any discrepancies through discussion until agreement was reached on the label that best represented the content of each cluster.

**Table 2:** Lemmas characterizing the three clusters identified by CA.

Cluster 1		Cluster 2		Cluster 3	
Material and family stability		Individual self-development		Connection and Social Realization	
Lemmas	Frequency	Lemmas	Frequency	Lemmas	Frequency
Economical	70	Limits	24	Dedicating	14
Relationship	37	Psychic	20	Growing	21
Tranquility	18	Physical	53	Capacity	31
Family	80	Harmony	17	Surrounding	18
Individual	19	Consequence	13	Caring	19
Good relationships	11	Goal	16	Friends	22
Safety	16	Social relations	16	Sharing	9
Physical health	17	Change	11	Close	9
Factor	25	Arrive	6	Time	25
Home	9	Satisfy	21	Believing	16

Χ^2^: Chi-square value. The table shows the first 10 words that characterize the single cluster.

Then, a correspondence analysis (COR) was performed on the lemma-cluster matrix to draw the factorial structure of the corpus (Greco and Polli, 2020). The COR breaks down and reorganizes the relationships between lemmas within a factorial space in which each dimension is composed of two polarities. Each polarity is characterized by a set of lemmas that tend to co-occur, and for each of these, the COR extracts an estimate (expressed through Chi-square scores) of the association between the factorial dimensions and the lemmas that characterize them. These estimates are used to interpret each polarity of the extracted factorial dimensions (Table 3).

**Table 3:** Lemmas characterizing the two factors extracted by COR.

First factor				Second factor			
Well-being				Conditions			
Individual (−)		Social (+)		Constructed (−)		Given (+)	
Lemmas	Contr.	Lemmas	Contr.	Lemmas	Contr.	Lemmas	Contr.
Limits	4.03%	Family	1.46%	Cultivate	2.82%	Economic	4.11%
Mental	2.99%	Friend	1.35%	Dedicate	2.62%	Relationship	2.81%
Physical	1.89%	Situation	1.10%	Capacity	2.42%	Serenity	2.55%
Harmony	1.67%	Respect	1.02%	Care	1.60%	Individual	2.54%
Consequence	1.63%	Colleagues	0.98%	Search	1.50%	Tranquillity	2.17%
Objective	1.51%	Ours	0.93%	Time	1.45%	Good relationships	1.76%
Social relations	1.32%	Work	0.85%	Surround	1.27%	Physical health	1.56%
Change	1.26%	Dear	0.84%	Interests	1.26%	Environment	1.42%
Arrive	1.20%	Professional	0.83%	Share	1.18%	Health	1.41%
Achieve	1.20%	See	0.72%	Close	1.18%	Home	1.35%

Contr: contribution (−): negative pole, (+): positive pole.

In sum, cluster analysis detects thematic representations whereas correspondence analysis discloses the symbolic dimensions that organizing discourse at a deeper level representing the latent oppositional tensions that polarize the narrative space (Factors) (Di Trani et al., 2021; Greco and Polli, 2020).

To assess the statistical adequacy of the corpus, the type/token ratio (acceptable for values < 0.20) and the percentage of hapax (considered adequate for values around 50%) were checked, as suggested by Bolasco (1999) and Greco (2016).

Finally, chi-square tests were performed to verify the association between clusters and the occupational variables (age, current role, flight hours).

## 3 Results

The textual corpus was statistically processable, as evidenced by a type/token ratio of less than 0.20 (0.16) and a hapax percentage of around 50% (0.57).

The CA conducted on the corpus identified three clusters as the optimal partition (Table 2). These clusters were subsequently interpreted by the research team as follows:

### 3.1 Cluster 1: material and family stability (41% elementary contexts classified)

This cluster is characterized by lemmas associated with economic security (economic), stable relationships (relationship), and overall tranquillity (tranquillity). Additionally, references to family, individual well-being, and good relationships indicate a strong emphasis on stability within both personal and familial contexts. The presence of terms such as safety, physical health, and home further reinforces the centrality of material security and familial cohesion in this thematic grouping. Illustrative excerpts of this cluster taken from participants’ responses are: *“The concept of a person’s well-being is primarily understood as the economic and financial aspect of an individual’s life,”* “*Well-being in life is represented by a harmonious family environment, a good relationship with one’s partner and children, financial stability*”.

### 3.2 Cluster 2: individual self-development (36% elementary contexts classified)

This cluster reflects a focus on personal growth and psychological balance, as indicated by the prominence of terms such as limits, psychic and physical. The lemma harmony suggests an aspiration for inner balance, while goal and change highlight an orientation toward self-improvement and adaptability. Moreover, including social relations and satisfy suggests that personal development is perceived in connection with social well-being. Illustrative excerpts of this cluster taken from participants’ responses are: *“In my opinion, a person’s well-being consists of the physiological and psychological balance of the individual — someone who is calm and satisfied with who they are and what they do, with meaningful social and emotional relationships, and in good physical health,” “Awareness of one’s own abilities and limitations, having a positive and flexible long-term outlook in relation to changes as well as good social relationships and, in general, a healthy relationship with one’s body and self”.*

### 3.3 Cluster 3: connection and social realization (23% elementary contexts classified)

The third Cluster centres on relational fulfillment and social engagement. Key terms such as dedicating, growing, and capacity indicate an active pursuit of personal and interpersonal development. The presence of surrounding, caring, and friends underscores the importance of relationships and social support. Furthermore, concepts such as sharing, close, and believing suggest that connection with others is fostered through shared experiences, trust, and emotional investment.

Illustrative excerpts of this cluster taken from participants’ responses are: *“Serenity, care in what one does and says, and the ability to dedicate time to meaningful relationships and personal interests,” “Accepting oneself for who one is and striving to improve the aspects one does not like, while setting achievable goals,” “Having a good relationship and connection with oneself by accepting both strengths and weaknesses, having someone by your side to fully share your life journey, and finding time to pursue your passions and interests”.*

The COR extracted two Factors that explained 55.37 and 44.63% of the variance, respectively. The characteristic lemmas of the two Factors (Table 3) allowed us to interpret these dimensions in the following way:

First Factor Individual well-being vs. social well-being. The first Factors is structured around a contrast between a concept of well-being centered on the individual (negative pole) and one oriented toward the social dimension (positive pole). The lemmas characterizing the negative pole, such as limits (contribution = 4.03%), mental (2.99%), and physical (1.89%), refer to a perspective in which well-being is conceived in terms of self-regulation, psychophysical balance, and management of one’s conditions. On the contrary, the positive pole is characterized by lemmas such as family (1.46%), friend (1.35%), and situation (1.10%), which suggest an interpretation of well-being as the result of interpersonal relationships, social support, and integration into the surrounding context. Furthermore, terms such as respect (1.02%) and colleagues (0.98%) reinforce this vision, indicating the role of work and social interactions in constructing well-being.

Second Factors Constructed conditions vs. given conditions. The second Factor reflects two different ways of conceiving the achievement of well-being. The negative pole is dominated by keywords such as cultivate (2.82%), dedicate (2.62%), and capacity (2.42%), which indicate a vision in which well-being is the result of an active process of personal construction. The implication of the subject also emerges through lemmas such as care (1.60%), search (1.50%), and interests (1.26%), which emphasize the necessity of a direct commitment in the search for one’s own realization. On the contrary, the positive pole is characterized by lemmas such as economic (4.11%), relationship (2.81%), and serenity (2.55%), which suggest an idea of well-being as a condition pre-existing or guaranteed by external factors. Lemmas such as physical health (1.56%), environment (1.42%), and home (1.35%) confirm this interpretation, highlighting the importance of structural and material conditions in the perception of well-being.

**Figure 1:**
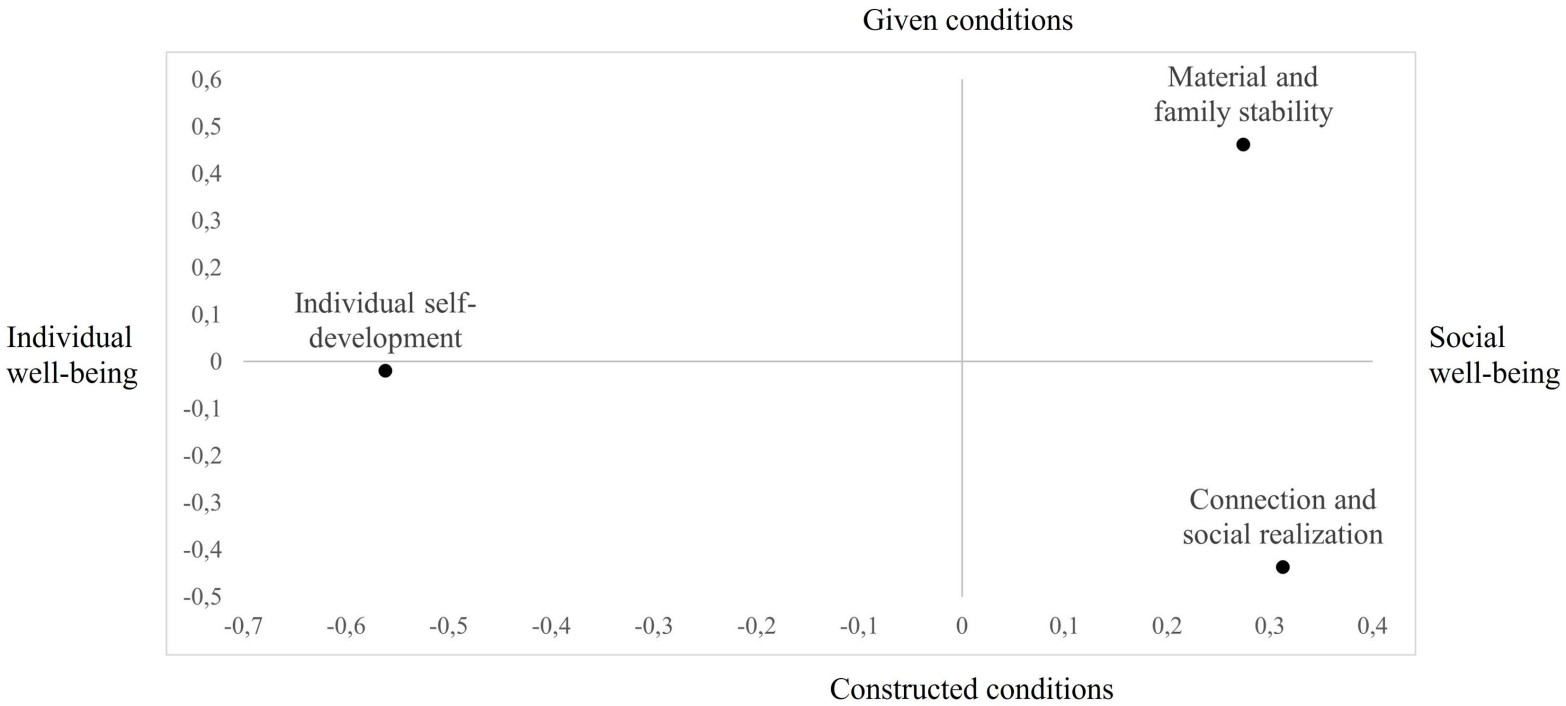
Positioning of the clusters in the space defined by the factors.

The representation of the Clusters in the factorial space (Figure 1) highlights their distribution along the two Factors. Precisely, the first Cluster (Material and family stability) is positioned in the positive polarity of the first Factor (Social well-being) and the positive polarity of the second Factor (Given conditions). This position suggests that well-being is conceived as the result of external and material conditions already available rather than as a process to be actively constructed.

The second Cluster (Individual self-development) is at the negative end of the first Factor (Individual well-being) and is almost neutral concerning the second Factor. This position indicates a concept of well-being centered on the individual and his or her personal growth, with less emphasis on social or material conditions.

The third Cluster (Connection and social realization) is positioned in the positive polarity of the first Factor (Social Well-being) but in the negative polarity of the second Factor (Constructed conditions). Unlike Cluster 1, here, well-being is conceived as the result of an active investment in time and interpersonal relationships.

Regarding the second aim of the study, the chi-square tests conducted to verify the association between the Clusters and the professional variables (gender, current role, flight hours) did not show statistically significant associations.

## 4 Discussion

The primary objective of this study was to explore the representations of well-being among crew members through qualitative approaches that allow for the identification of how well-being is emotionally symbolized within this professional category. This exploration aims to uncover elements that may aid in understanding the willingness of crew members to engage with the “Support Program”.

In our study emerges a factorial space defined by two Factors: one contrasting individual and social representations of well-being, and the other opposing well-being as a sum of given elements (e.g., economic stability, relationships, serenity) to well-being as a dynamic and ongoing process requiring active engagement through actions and choices (e.g., cultivation, dedication, capacity). These polarities organize space by linking positions ranging from self-oriented to socially oriented dimensions, from more passive/concrete conceptions to more active/purpose-driven ones.

Interestingly, as shown in Figure 1, not all the space defined by the two Factors is occupied by the emerged clusters. Specifically, regarding the polarity of individual well-being, only one Cluster (2) is present, which is not influenced by the second Factor. Cluster 2, the largest one, defines well-being as a highly individual concept, focusing on personal growth and psychological balance, and emphasizes the perception of having achieved harmonious development and mental and emotional stability. The polarity of social well-being contains two Clusters, both influenced by the second Factor. Cluster 1, the second-largest one, is between social well-being and given conditions. It represents well-being as the sum of socially desirable elements such as economic stability, having a family, feeling calm, being employed, and maintaining good health. The social dimension here seems to be meant as “social desirability.” In contrast, Cluster 3, the smallest one, is positioned between social well-being and constructed conditions. The most frequently mentioned words in this Cluster are “dedicate” and “cultivate,” indicating a view of well-being as actively developed through care, friendships, and emotional sharing. In this Cluster, the social dimension is seen as a resource. In summary, three central representations of well-being emerged, showing a progression from external/concrete dimensions (material elements) to more internal/intrapsychic ones—shifting from the intrapersonal (e.g., possessing status symbols) to the interpersonal, focusing on personal responsibility in achieving well-being. An interesting finding is that work is only marginally represented in well-being representations. A connection to work appears only in the first Cluster (as thirteen lemmas, and therefore do not appear in Table 2, which is limited to reporting only the top ten lemmas), focusing on material and concrete aspects of well-being. This suggests that work is seen more in terms of its economic value/status than as an expression of personal fulfillment. The idea that there could be a condition of well-being connected to the workplace seems to be missing.

In closed questions analysis (see Reho et al., 2025), on the other hand, only two out of five US recognized the importance of psychological well-being. However, they did not show any willingness toward the “Support Programme,” fearing a negative impact on their work. A defensive attitude, therefore, emerges that tends to disconnect the theme of well-being/illness from the work.

Another interesting consideration is the relatively low response rate to the open question on well-being (299 out of 791 respondents chose not to complete it). This may be attributed to two main factors: (1) difficulty fully engaging with the questionnaire requests showing a more personal dimension of well-being, and (2) fear of judgment or potential repercussions in the work context, which may have heightened social desirability bias. This aligns with Cross et al.’s (2024) findings, highlighting that pilots are reluctant to discuss mental health issues due to fears of negative consequences, often doubting the confidentiality of reporting systems. Our findings lead us to speculate that the willingness of flight crews to use the “Support Programme” may be compromised by fear of job repercussions. This recommends the need for interventions aimed at fostering a cultural shift that cultivates a perception of the work environment that is less threatening and more trustworthy. As suggested by Nielsen et al. (2010), in fact, the success of a project, even if aimed at promoting staff well-being, requires several elements facilitating its acceptance and development. In particular, it seems worthwhile to us to remember three elements among those indicated by the authors: (a) interventions should prioritize organizational-level strategies (primary interventions) to improve the design, structure, and management of work; (b) active employee participation should be a fundamental aspect of these interventions; (c) implementation should consider how occupational health programs can be effectively integrated into existing organizational procedures, workplace culture, and overall occupational safety and health management. In this regard, the present study appears to highlight low employee participation and limited integration into workplace culture as weaknesses of the “Support Programme.” It is therefore essential that targeted policy actions be implemented to address these issues, in order to promote greater use of the programme, enhance crew members’ well-being, and ultimately improve passenger safety. To achieve this, and in line with Nielsen et al. (2010) it is crucial to implement policy interventions that not only reorganize the structural and functional aspects of the organization, but also challenge its cultural dimensions. In this regard, the use of Participatory Action Research (PAR) models may be particularly effective, as this approach actively involves the recipients of the intervention in transforming their reference context (Stern, 2019).

### 4.1 Limitations

First, the gender distribution was unbalanced, highlighting the need for more representative samples thus required in future studies. Second, only Italian participants were included; future research should expand to aviation workers from other countries to explore specific cultural differences. The work-related context may have increased social desirability bias, potentially affecting responses. Furthermore, the adopte consensus-based labeling approach inherently relies on the researchers’ subjective judgment, potentially introducing bias in cluster characterization. Additionally, the absence of quantitative inter-rater metrics limits the assessment of labeling consistency across raters. Future studies could adopt integrated approaches, combining quantitative metrics (e.g., Cohen or Fleiss kappa) to assess agreement between raters with computational techniques (such as topic modeling or automatic keyword extraction), thereby reducing subjectivity in labeling.

Moreover, future studies may consider exploring this theme more broadly in the general population by retrospectively applying job-related categories to participants, in order to help reduce social desirability bias linked to the context of data collection. The snowball sampling method also introduced self-selection bias, limiting sample representativeness.

## 5 Conclusion

Despite these limitations, the findings offer valuable insights into the cultural representation of well-being among pilots and cabin crew, allowing for the identification of three principal emotional symbolizations of this concept within this specific occupational group. Nevertheless, these representations did not appear to be associated with specific job dimensions, suggesting a disconnection between the concept of work and that of well-being. Given the lack of studies in this population, mainly using a quantitative-qualitative approach, these results could inform organizational policies and awareness campaigns promoting support programs. The workplace, where employees spend significant time, provides an ideal setting for health promotion interventions to improve well-being (Poscia et al., 2016).

## Data Availability

The raw data supporting the conclusions of this article will be made available by the authors, without undue reservation.
